# Energy Transfer between Tb^3+^ and Eu^3+^ in LaPO_4_: Pulsed versus Switched‐off Continuous Wave Excitation

**DOI:** 10.1002/advs.201900487

**Published:** 2019-04-05

**Authors:** Yuxia Luo, Zhenyu Liu, Hon Tung Wong, Lei Zhou, Ka‐Leung Wong, Kwok Keung Shiu, Peter A. Tanner

**Affiliations:** ^1^ Department of Chemistry Hong Kong Baptist University 224 Waterloo Road Kowloon Hong Kong S.A.R., P. R. China; ^2^ Hong Kong Baptist University Institute of Research and Continuing Education Shenzhen Virtual University Park Shenzhen P. R. China; ^3^ MOE Key Laboratory of Bioinorganic and Synthetic Chemistry, School of Chemistry Sun Yat‐sen University Guangzhou 510275 P. R. China

**Keywords:** energy transfer, lanthanide, migration, pulse and continuous wave excitation

## Abstract

The energy transfer (ET) between Tb^3+^ and Eu^3+^ is investigated experimentally and with available theoretical models in the regime of high Tb^3+^ concentrations in ≈30 nm LaPO_4_ nanoparticles at room temperature. The ET efficiency approaches 100% even for lightly Eu^3+^‐doped materials. The major conclusion from the use of pulsed laser excitation and switched‐off continuous wave laser diode excitation is that the energy migration between Tb^3+^ ions, situated on La^3+^ sites with ≈4 Å separation, is not fast. The quenching of Tb^3+^ emission in singly doped LaPO_4_ only reduces the luminescence lifetime by ≈50% in heavily doped samples. Various theoretical models are applied to simulate the luminescence decays of Tb^3+^ and Tb^3+^, Eu^3+^‐doped LaPO_4_ samples of various concentrations and the transfer mechanism is identified as forced electric dipole at each ion.

## 1 Introduction

Lanthanide ions present unique spectral properties, including long lifetime, multiple narrow and well‐separated bands, and large effective shifts from excitation to emission wavelength. They can be model candidates as donor and acceptor in the study of energy transfer (ET). Hence, there have been a plethora of ET research studies concerning lanthanide ions, such as between the tripositive ions Ce‐Eu,^1^ Ce‐Tb,^2^ Tb‐Eu,^3^ Pr‐Yb,^4^ Dy‐Tb,^5^, ^6^ Sm‐Eu,^6^, ^7^, ^8^ and Ho‐Yb,^9^, ^10^ with the focus upon the applied optical properties of phosphors, such as the color tunability of luminescence and optical thermometry. However, Dutra et al.^11^ have pointed out that in 2015 less than 3% of published studies concerning lanthanide ions made use of theoretical tools. In particular, the ET between Tb^3+^ and Eu^3+^ has attracted much attention (Table S4, Supporting Information). The ^5^D_4_ donor state emission from Tb^3+^ is quenched and instead red emission from the Eu^3+ 5^D_0_ state is observed when exciting into a Tb^3+^ absorption band. The detailed energy transfer mechanism is not clear in many cases. For example, Moran et al.^12^ studied the cubic system Cs_2_NaYCl_6_ doped with Tb^3+^ and Eu^3+^ in a wide range of concentrations at 298 and 77 K. It was concluded that discrepancies between the calculated and experimental rate parameters are large, notably by four or five orders of magnitude for multipole–multipole mechanisms. Whereas it could be possible that different interaction mechanisms take part in different solid‐state lattices, various studies have attributed the interaction mechanism between Tb^3+^ and Eu^3+^ to electric dipole–electric dipole (ED–ED),^13^, ^14^, ^15^, ^16^ or electric dipole–electric quadrupole (ED–EQ)^17^ or exchange interaction.^18^, ^19^ The ^5^D_4_ →^7^F_*j*_ (*J* = 6‐4) transitions of Tb^3+^ are forced ED allowed whereas the ^5^D_0_ ←^7^F_0_ transition of Eu^3+^ is dipole forbidden,^20^ but at room temperature the ^5^D_0_ ←^7^F_1_ forced ED pathway is also available. The term forced (or induced) ED refers to 4f–4f transitions being enabled via the wavefunction mixture with 5d (or higher) opposite‐parity configurations through the crystal field symmetry or vibrations of the appropriate symmetry. Furthermore, the energy migration between the donor Tb^3+^ ions has been stated as very fast^21^ or slow.^22^ For example, Blasse^23^ envisaged that the migration rate between lanthanide ions such as Eu^3+^ or Gd^3+^ was up to 10^7^ s^−1^ for a separation of 4 Å, whereas Dornauf and Heber^24^ found negligible concentration quenching of Tb^3+^ in LaP_5_O_14_. The process of migration between identical ions leads to an increase in lifetime, whereas termination of the process at a trap leads to a decrease.

**Figure 1:**
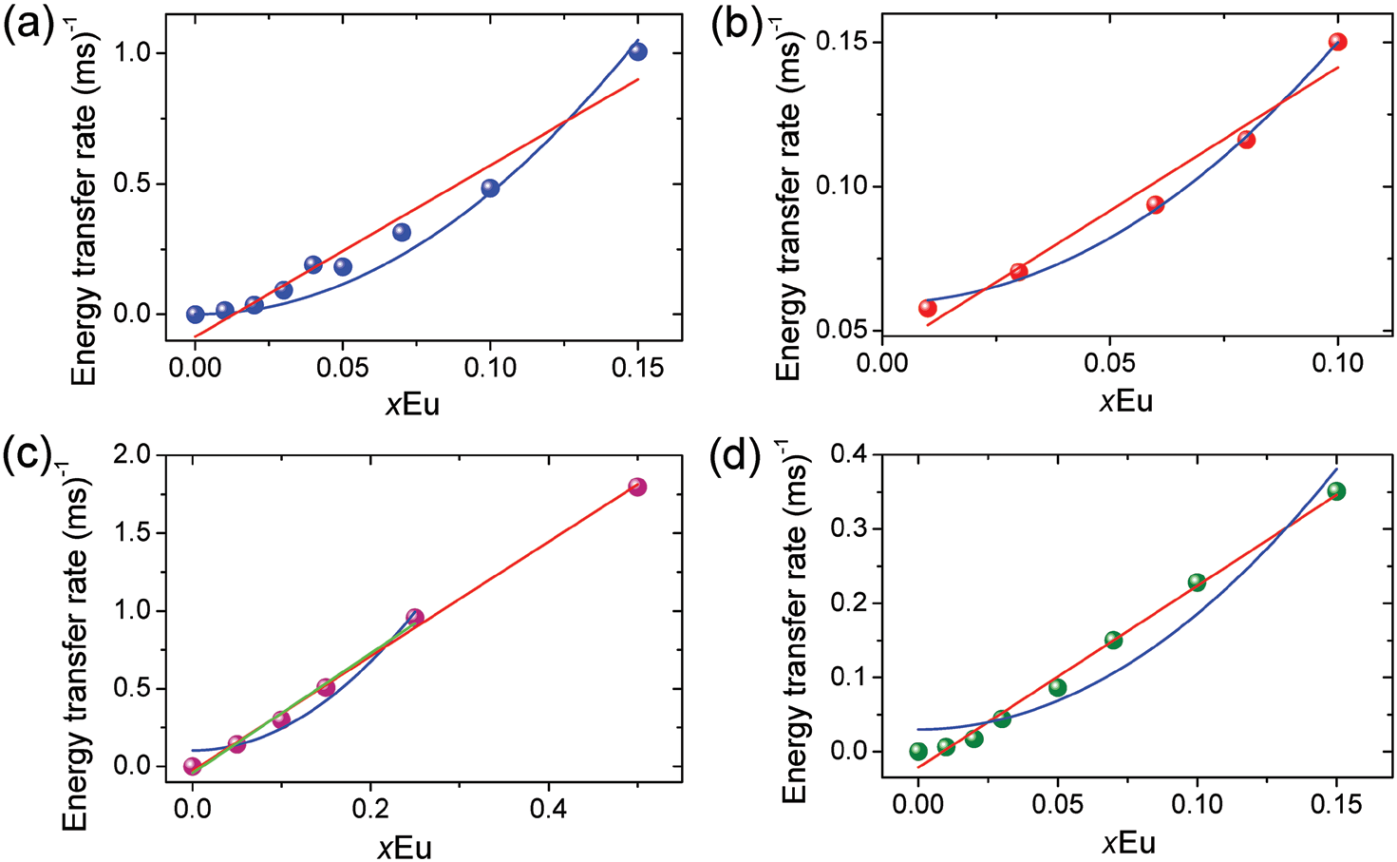
Tb^3+^‐Eu^3+^ energy transfer rates for some systems with low Eu^3+^ concentrations from literature data, fitted by *y* = *A* + *Bx* ^2^ (blue lines) and *y* = *C* + *Dx* (red lines): a) Cs_2_NaTb_1−_ *_x_*Eu*_x_*Cl_6_ at 293 K, ^[27]^ *A* = 0.065 ± 0.004, *B* = 42.7 ± 0.04, $R_{\mathrm{adj}}^{2}$ = 0.9930; b) TlY_2.25−_ *_x_*Tb_0.75_Eu*_x_*F_10_ at 295 K,^[28]^ calculated from lifetime data therein and fitted by: *A* = 0.0596 ± 0.0017, *B* = 9.0 ± 0.3, $R_{\mathrm{adj}}^{2}$ = 0.9956; c) Cs_2_NaY_0.995−_ *_x_*Tb_0.005_Eu*_x_*Cl_6_ at 295 K,^[12]^ *A* = 14.28 ± 1.7, *B* = 0.10 ± 0.05, $R_{\mathrm{adj}}^{2}$ = 0.948; d) Cs_2_NaTb_1−_ *_x_*Eu*_x_*Cl_6_ at 80 K,^[24]^ *A* = 15.6 ± 1.6, *B* = 0.03 ± 0.01, $R_{\mathrm{adj}}^{2}$ = 0.928. The linear fittings are shown in red and in green for the reduced range of *x*Eu in (c). Note the superior quadratic fittings in (a), (b) and the linear fits in (c), (d).

The kinetics of an ET process as a function of concentration have been studied by several authors, for example, by Fong and Diestler^25^ and Lupei.^26^ A linear relation between the energy transfer rate and acceptor concentration has been taken to indicate a two‐ion process, whereas a quadratic relation is indicative of a three‐ion process. Lupei^26^ studied the quenching of Er^3+4^S_3/2_ emission in YAG: Er^3+^ at high concentrations of Er^3+^ and explained the variation of the energy transfer rate in terms of a three‐body process where two acceptor ions are promoted from the electronic ground state to ^4^I_9/2_ and ^4^I_13/2_ *J*‐multiplets. Hence, we were very surprised to observe the same type of interaction in two literature studies of Tb^3+^‐Eu^3+^ ET^27^, ^28^ (**Figure** **1**a,b) where Eu^3+^ concentration is varied when Tb^3+^ concentration is high: for Cs_2_NaTb_1−_ *_x_*Eu*_x_*Cl_6_ at 293 K and TlY_2.25−_ *_x_*Tb_0.75_Eu*_x_*F_10_ at 295 K. However, at higher Eu^3+^ concentrations in Cs_2_NaY_0.995−_ *_x_*Tb_0.005_Eu*_x_*Cl_6_ at 295 K and Cs_2_NaTb_1−_ *_x_*Eu*_x_*Cl_6_ at 80 K (Figure 1c,d), the variation of energy transfer rate with Eu^3+^ concentration is clearly linear.

**Figure 2:**
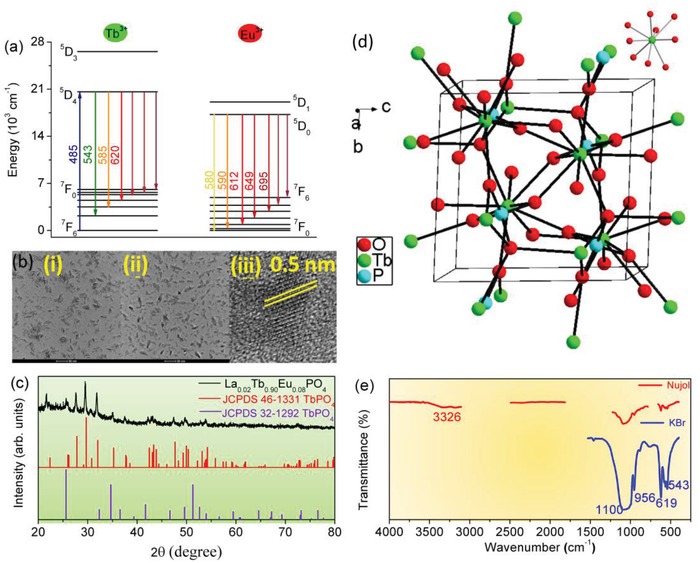
a) Relevant electronic energy level schemes of 4f^8^ Tb^3+^ and 4f^6^ Eu^3+^. The overall ET process involves the depopulation of Tb^3+5^D_4_ and excitation of Eu^3+ 5^D_0_. b) TEM of nanocrystals: La_0.10_Tb_0.90_PO_4_ (i), La_0.02_Tb_0.90_Eu_0.08_PO_4_ (ii) (the scale is 50 nm), and HRTEM showing individual nanocrystals of the latter (iii); c) XRD patterns for La_0.02_Tb_0.90_Eu_0.08_PO_4_ and the JCPDS files: No. 46‐1331 of monoclinic and 32‐1292 of tetragonal TbPO_4_ for comparison; d) crystal structure of TbPO_4_; the inset depicts the coordination geometry of Tb^3+^; e) FTIR spectrum of nanocrystals La_0.02_Tb_0.90_Eu_0.08_PO_4_ dispersed in Nujol (the bands of Nujol at 2925, 2856, 1460, 1377, and 723 cm^−1^ were subtracted) and KBr, respectively.

These unusual findings prompted us to reinvestigate the rate dependence of Tb^3+^‐Eu^3+^ ET as a function of concentration and demonstrate that in fact it is a two‐body process and not a three‐body process as proposed elsewhere.^20^, ^27^ We selected the lanthanide orthophosphate host because of its high insolubility, non‐hygroscopicity, reliable stability against high temperature, low toxicity, and high quantum yield.^14^ Naturally, it is a prerequisite to determine the photophysics of the singly doped host prior to an ET study involving two lanthanide ions, and we have performed this for LaPO_4_ doped with Tb^3+^. Whereas most previous studies have utilized micrometer‐sized samples synthesized at high temperature (Table S4, Supporting Information), we prepared nanomaterials doped with Tb^3+^ and Eu^3+^ because the occurrence of trap species, if important, would then be magnified in our study. We take the previous study of Chen and co‐workers^29^ on similar size nanoparticles of Eu^3+^‐doped hexagonal hydrated TbPO_4_ as a reference for comparison of our results obtained from nanomaterials prepared by a modified method of Haase and co‐workers.^30^, ^31^ The relevant energy level schemes of Tb^3+^ and Eu^3+^ are displayed in **Figure** **2**a.

The major aims of our study were to investigate (i) the importance of energy migration between terbium ions in the LaPO_4_ host, where the donor–donor separation is about 4 Å; and (ii) to give a quantitative description of Tb^3+^‐Eu^3+^ ET in this host lattice, where complications such as charge inequality and multisite occupation of the ions are absent. With the use of different excitation techniques and various theoretical models, these aims have been fully addressed.

## 2 Theoretical Section

Various models have been employed to simulate the donor decay after pulsed excitation when energy is transferred from a donor to acceptor ions in order to ascertain the interaction mechanism. We largely focus upon ED–ED energy transfer because the relevant lanthanide ion transitions are of forced electric dipole character. The donor emission intensity at time *t*, relative to the initial intensity, when in the presence of an acceptor, is given by^32^

(1) $$I_{D}\left(t\right)=I_{D}\left(0\right)\exp\left[-\left(\left[1/\tau_{D}\right]+k_{\mathrm{ET}}\right)t\right]$$

where τ_D_ is the lifetime of donor in the absence of the acceptor and *k*_ET_ is the energy transfer rate (=*P*_DA_ *c*_A_, where *P*_DA_ is a parameter describing the donor–acceptor interaction and *c*_A_ is the acceptor concentration). This equation holds for an isolated donor–acceptor pair, or for an acceptor in an average donor environment due to rapid energy migration between donors. In the absence of donor–donor migration, the decay is not monoexponential because the acceptors at sites *q* are situated at different distances^32^, ^33^

(2) $$I_{D}\;\left(t\right)=I_{D}\;\left(0\right)\;\exp\left\{-\left(\frac{1}{\tau_{D}}\right)\;\prod_{q}\left[1-c_{A}+c_{A}\exp\left(-X_{0q}t\right)\right]\right\}$$

where the sum over *X*_0_ *_q_* represents the total donor–acceptor transfer rate. In the continuum approximation (which predicts excessive energy transfer rates at very small donor–acceptor separations), the Inokuti–Hirayama model^34^ for multipole interactions assumes a random arrangement of acceptors and the intensity of emission at time *t* is related to that initially by

(3) $$I_{D}\;\left(t\right)=I_{D}\;\left(0\right)\exp\left\{-\frac{t}{\tau_{D}}-\Gamma \left(1-\frac{3}{s}\right)\frac{c_{A}}{c_{0}}{\left[\frac{t}{\tau_{D}}\right]}^{3/s}\right\}$$

where *s* = 6, 8, or 10 according to the electric multipolar mechanism ED–ED, ED–EQ, or EQ–EQ, respectively; Γ is the gamma function;^34^ *c*_A_ = (*x* × *Z*)/*V*), where *x* is the stoichiometric mole fraction, *Z* is the number of acceptors in the unit cell, and *V* is its volume; *c*_0_ is the critical (reduced) acceptor concentration

(4) $$c_{0}=\frac{3}{4\pi R_{0}^{3}}$$

where *R*_0_ is the critical distance. Instead, if transfer is considered to shells of acceptor ions in a crystal^24^

(5) ID (t)=ID (0)exp{−tτD}exp{−4πR0scAtτD(s−3) k+1Rs−3}[∑l = 1kZlZ exp{−tτD(R0Rl)s}]Zx

where *^l^Z* is the total number of acceptor sites present in shell *l* at distance *^l^R* from the donor; *Z* is the total number of sites in *k* shells; and *x* is the acceptor molar concentration. *N* is the total number of acceptors = *Zx*. The second exponential function represents a correction factor for acceptors outside the chosen shells. The other symbols are defined above.

An alternative description of the shell model for small (≈3 nm) spherical nanoparticles, based upon the master Equation (2), and correcting for the proximity of the donor to the surface, has been given by Rabouw et al.^35^, ^36^ This equation is not necessary in the present case since our particles are rather larger.

In Equations (2), (3), and (5), no account is taken of donor–donor migration or donor–acceptor back transfer. The presence of 3D energy migration may be examined by the analysis of the (monoexponential) donor decay lifetime, τ, at very long times after the excitation pulse, the asymptotic behavior (*t* → ∞)

(6) $$I_{D}\left(t\right)=I_{D}\left(0\right)\exp\left[-\left(t/\tau \right)\right],\;\;\mathrm{where}\;\;\frac{1}{\tau }=\frac{1}{\tau_{D}}\;+\frac{1}{\tau_{\mathrm{Df}}}$$

where, in this case, τ_D_ represents the intrinsic donor lifetime and for weak diffusion (when the donor–donor migration rate is smaller than the donor–acceptor transfer rate), with the rate *k*_Df_ = 1/τ_Df_

(7) $$\;\frac{1}{\tau_{\mathrm{Df}}}=\;4\pi c_{A}D\rho \;\;\mathrm{and}\;\;\rho \;=\;0.68{\left(C/D\right)}^{1/4}$$

Here, *D* is a diffusion constant and *C* is a donor–acceptor interaction constant. Alternatively, for weak diffusion, the simplest equation for the inclusion of migration between donor ions, for the case of ED–ED interaction is^37^

(8) $$I_{D}\;\left(t\right)=I_{D}\;\left(0\right)\exp\left\{-\frac{t}{\tau_{D}}-\frac{4}{3}\pi^{\frac{3}{2}}c_{A}{\left\{\alpha_{\mathrm{DA}}^{\left(6\right)}t\right\}}^{1/2}{\left[\frac{1+a_{1}x+a_{2}x^{2}}{1+bx}\right]}^{3/4}\right\}$$

where *a*_1_ = 10.866, *a*_2_ = 15.500, *b* = 8.743; $x\;=\;D{\left\{\alpha_{\mathrm{DA}}^{\left(6\right)}\right\}}^{-1/3}t^{2/3}$. *D* is a diffusion constant and $\alpha_{\mathrm{DA}}^{\left(6\right)}$ is the ED–ED interaction parameter for donor–acceptor transfer $\left(=R_{0}^{6}/\tau_{R}\right)$. Analogous equations have been given for donor–acceptor exchange interaction, and for the occurrence of migration for types of interaction other than ED–ED, and the reader is referred to the original publications for details.^24^, ^34^, ^37^, ^38^ In principle, the fitting of donor decay by these equations yields the interaction mechanism, the critical distance (i.e., when the rate of ET is equal to the donor decay rate), and the magnitude of the diffusion constant. When the donor–donor migration rate exceeds that of donor–acceptor transfer, a random hopping model is more appropriate.^39^

The above equations refer to the donor emission profile after a short excitation pulse. The donor emission decay profile following switched‐off continuous excitation has been discussed by Eisenthal and Siegel^40^ and Siebold and Heber.^41^ Pulsed excitation provides an instantaneous population of randomly excited donors at *t* = 0 (cf. Equation (3)) with the acceptor ions unexcited. By contrast, switched‐off continuous excitation gives the steady‐state population of excited donors. In this case, the excited donors are not randomly distributed—the probability of an excited donor being near an acceptor is smaller than that of an excited donor with an acceptor further away. The donor decay will therefore differ from the case of pulsed excitation *unless* very rapid migration of energy occurs between the donors. Assuming a random population of unexcited donors and acceptors, in the absence of donor–donor migration, the donor decay at time *t*, after switched‐off continuous excitation at time *t* = 0, can be represented in the case of ED–ED transfer by^40^

(9a) $$I\;\left(t\right)=\;I\left(0\right)\left[\exp\left(-\frac{t}{\tau }-2q{\left[\frac{t}{\tau }\right]}^{\frac{1}{2}}\right)-\pi^{1/2}q\;\exp\left(q^{2}\right)\left\{1-\mathrm{erf}\left[q+{\left[\frac{t}{\tau }\right]}^{\frac{1}{2}}\right]\right\}\right]$$

where

(9b) $$q\;=\;\left(N_{A}/2\right)\left(R_{0}^{3}/R_{v}^{3}\right)\;\pi^{\frac{1}{2}}=\;\left(\pi^{\frac{1}{2}}/2\right)\left(c_{A}/c_{0}\right)$$

and

(9c) $$\mathrm{erf}\left(x\right)=\frac{2}{\pi^{1/2}}\;\int_{0}^{x}\exp\left(-y^{2}\right)dy$$

Here, τ is the donor lifetime in the absence of acceptors; *N*_A_ is the number of unexcited acceptors; *R*_0_ is the critical transfer distance for which the probability of nonresonance deactivation and resonance transfer is equal for a donor–acceptor pair; and *R*_v_ is the vessel radius. The donor decay under the same conditions for pulsed excitation is given by Equation (3).

## 3 Results and Discussion

### 3.1 Structure and Morphology of the Crystals

The morphology of La_0.10_Tb_0.90_PO_4_ (i) and La_0.02_Tb_0.90_Eu_0.08_PO_4_ (ii) nanocrystals, together with the high‐resolution transmission electron microscopy (HRTEM) image of the latter (iii) are presented in Figure 2b. The nanocrystals are elongated compared with the size of the LaPO_4_ nanocrystals prepared by Haase and co‐workers,^31^ which is due to the decreasing amount of the complexing agent triethyl phosphate employed.^30^ The HRTEM image displays lattice fringes with spacing 0.5 nm for most particles which indicates a highly crystalline material. The measured average size of the nanocrystals is 30 nm compared with that of 34 nm determined from the line broadening in the X‐ray diffraction (XRD) pattern (Figure 2c). The XRD pattern is consistent with the standard monoclinic card of TbPO_4_ with a shift of the peaks to lower angles since Eu^3+^ is a slightly larger ion than Tb^3+^. No additional peaks of other phases are present. The structure of the monoclinic form TbPO_4_ (space group *P*2_1_/*n*, *Z* = 4^42^) is shown in Figure 2d and is the same structure for the other lanthanide ions from La to Gd. The closest distance between the nine‐coordinate Tb^3+^ ions, with site symmetry *C*_s_, is 3.97 Å. There is another form of TbPO_4_ belonging to the tetragonal space group *I*4_1_/*amd* 43 with Tb^3+^ ions situated at sites of *D*_2d_ point group symmetry, with closest separation 3.79 Å.

The Fourier transform infrared (FTIR) spectra in Figure 2e show the characteristic phosphate group vibrations at (in cm^−1^): 543 (ν_4_), 956 (ν_1_), and ≈1086 (ν_3_).^44^ The band at 3326 cm^−1^ is characteristic of the O—H stretching vibration of H_2_O. The bands at 1457 cm^−1^ are due to the coating of TOA.

### 3.2 The Decay of Tb^3+^ Emission in LaPO_4_:Tb^3+^

**Figure 3:**
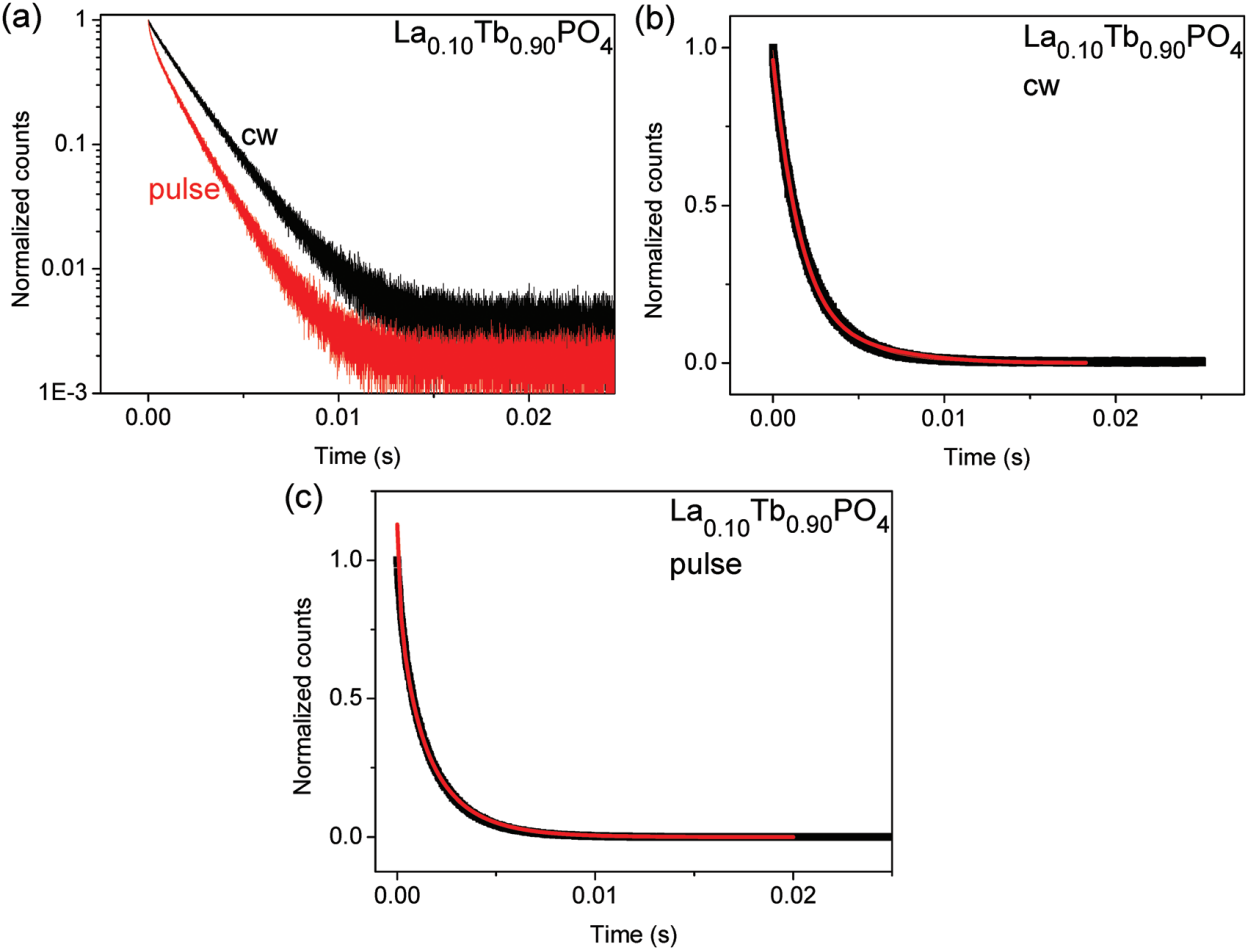
a) Room‐temperature luminescence decay curves of the ^5^D_4_ level in La_0.10_Tb_0.90_PO_4_ displayed on a logarithmic ordinate scale after switched‐off cw excitation and flash excitation; b,c) Fits of the curves on linear ordinate scales using Equations (9a) and (3), respectively.

The luminescence decay of the Tb^3+ 5^D_4_ state was monitored for LaPO_4_ doped with various concentrations of Tb^3+^, by exciting at 485 nm and collecting the emission of the ^5^D_4_ →^7^F_5_ transition at 543 nm. The decay curves for switched‐off continuous wave (cw) and for pulsed excitation are shown on a log scale in **Figure** **3**a for one sample of La_0.10_Tb_0.90_PO_4_ and show a distinct difference in the initial decay. The time width of the excitation pulse, in the microsecond regime, is therefore smaller than the energy migration hopping time. If the migration rate were fast, then the curves would be superimposable. The decay following cw excitation can be fitted by a monoexponential function in the region from 0 to 15 ms with the lifetime 1.556 ± 0.001 ms ($R_{\mathrm{adj}}^{2}$ = 0.99933) whereas that for pulsed excitation can be fitted by biexponential decay with the above fixed lifetime and another one of 0.258 ± 0.001 ms ($R_{\mathrm{adj}}^{2}$ = 0.99908). The decay after cw excitation represents the steady state of the migration and energy transfer to traps, whereas pulsed excitation provides an equal excitation probability for all donors. Hence, the difference could be due to the fact that some donors are near traps, that is, energy acceptors of a different species. The nature of the traps is subsequently discussed. The decays were fitted by Equation (9a) (for switched‐off cw excitation, Figure 3b, $R_{\mathrm{adj}}^{2}$ = 0.9992) and Equation (3) (for pulsed excitation, Figure 3c, $R_{\mathrm{adj}}^{2}$= 0.9984) using two variable parameters in each case. From these fits, the derived ratios of *c*_A_/*c*_0_ are 0.64 (cw) and 0.63 (pulsed), which give an acceptor concentration between 2.3 × 10^21^ and 4.5 × 10^19^ cm^−3^ for *R*_0_ values between 4 and 15 Å, respectively.

**Figure 4:**
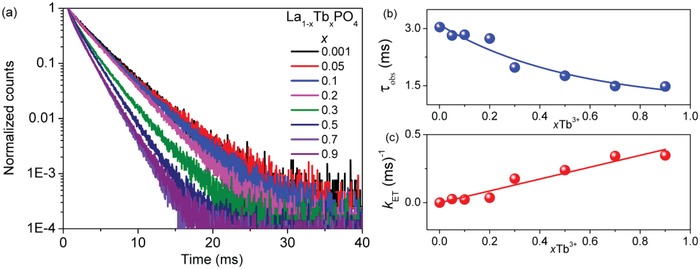
a) Luminescence decay curves; b) monoexponential lifetimes (τ_obs_); c) energy transfer rates (*k*_ET_) of La_1−_ *_x_*Tb*_x_*PO_4_ nanocrystals (λ_ex_ = 485 nm, λ_em_ = 543 nm).

The decay curves following pulsed excitation for Tb^3+^ in La_1−_ *_x_*Tb*_x_*PO_4_, after logarithmic transformation, are reasonably linear in the measured region from 0 to 20 ms, with $R_{\mathrm{adj}}^{2}$ > 0.998, except for La_0.10_Tb_0.90_PO_4_ with $R_{\mathrm{adj}}^{2}$ > 0.9963, as shown in **Figure** **4**a. These ^5^D_4_ lifetime data for all samples are summarized in Table S1 (Supporting Information) and demonstrate a monoexponential decrease in lifetime with increasing concentration of Tb^3+^ in La_1−_ *_x_*Tb*_x_*PO_4_ (Figure 4b). The lifetime of the La_0.10_Tb_0.90_PO_4_ nanocrystals (≈1.5 ms) is considerably longer than that reported at room temperature as 0.55 ms for TbPO_4_ nanowires (2 µm × 40 nm)^45^ and 0.98 ms for micrometer‐size TbPO_4_ 29 so that surface quenching in these latter two examples is clearly very important. The lifetime of a La_0.98_Tb_0.02_PO_4_ bulk sample^46^ was reported to be 3.1 ms which is similar to the measured lifetime of 3.04 ms for La_0.999_Tb_0.001_PO_4_ nanocrystals herein.

The use of Equation (1), where τ_D_ in this case is the lifetime at infinite dilution and *k*_ET_ is the ET rate to traps, gives a linear relation of *k*_ET_ with concentration (Figure 4c), which can be interpreted as showing that a two‐body process is responsible for quenching: Tb^3+^ – trap. The ET rate is slow: being only ≈350 s^−1^ for the La_0.10_Tb_0.90_PO_4_ sample. Recently, Johnson et al. have argued that migration to killer sites (i.e., nonradiative sinks) is more important than cross‐relaxation processes.^47^ This alternative quenching process: cross‐relaxation involving two or three^48^ Tb^3+^ ions is not possible when considering the Tb^3+^ energy level scheme in Figure 2a since the energy gap below ^5^D_4_ is nearly 15 000 cm^−1^. Fitting the long‐term decay curves with Equation (6) enables the determination of 1/τ_Df_, which is also not fast. This parameter gives a linear plot against Tb^3+^ concentration, as expected from Equation (7) (Figure S1, Supporting Information).

**Figure 5:**
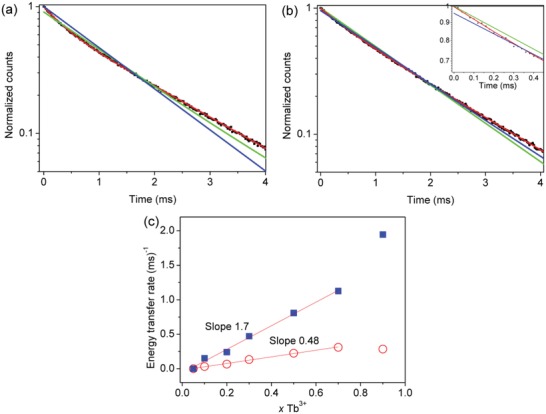
Fits of the decay of La_1−_ *_x_*Tb*_x_*PO_4_ samples by monoexponential (green) and biexponential (red) functions and Equation (3) (blue) for a) *x* = 0.90 and b) *x* = 0.70. The inset in (b) shows the initial decay to 0.5 ms. c) Energy transfer rates of Tb^3+^ ions with fast and slow decay. The fits are made for *x* = 0.05 up to *x* = 0.70. The values for *x* = 0.90 are not accounted for.

More detailed fits to the decay curves of La_1−_ *_x_*Tb*_x_*PO_4_ were then carried out. **Figure** **5**a,b shows the section of Tb^3+^ emission decay in the range from 0 to 4 ms with monoexponential (green), biexponential (red), and Inokuti–Hirayama, Equation (3) (blue), fittings for *x* = 0.9, 0.7. The Inokuti–Hirayama equation refers to energy transfer from a donor to a random array of acceptor ions without donor–donor migration. This model gives a poorer fit to the data. The biexponential fit is superior to the linear fit described above and was repeated for other Tb^3+^ concentrations with the results listed in Table S2 (Supporting Information). The biexponential fit is explained by the occurrence of two different environments of Tb^3+^ ions. In their study, van Hest et al.^46^ have associated two environments of Eu^3+^ in 3.9 nm nanocrystals of LaPO_4_ with surface (τ = 1.9 ms) and interior (τ = 4.7 ms) sites since the amplitudes if their fit were 44 and 56%, respectively. The occurrence of several sites for Eu^3+^ in La_0.98_Eu_0.02_PO_4_ has previously been reported from 77 K emission spectra by Dexpert‐Ghys et al.,^49^ who pointed out that in EuPO_4_ and LaPO_4_ the average Ln—O distances are 2.474 and 2.574 Å, respectively, so that in La_0.98_Eu_0.02_PO_4_ some distortion occurs. We find for the *x* = 0.05–0.70 samples of La_1−_ *_x_*Tb*_x_*PO_4_ that the mean amplitudes of the fast and slow components in the biexponential fits are about 32 and 68%, which differ considerably from the population values of about 4 and 96% for surface and interior ions in our nanoparticles. The energy transfer rate (*k*_ET_) for the ions decaying by the fast component is 3.6 times faster than for the slower‐decaying ions (Figure 5c). Our time‐resolved spectra following pulsed excitation do not show evidence from band positions or intensities, under the experimental resolution of several nm, for the assignment of spectral features to Tb^3+^ ions located at different sites. The faster decay is then associated with energy transfer to OH^−^ or H_2_O killer trap molecules (with the latter observed to be present from the FTIR spectrum) or other defect sites, followed by nonradiative decay. The slower decay is due to ^5^D_4_ emission at the regular Tb^3+^ C_s_ site in TbPO_4_.

In summary, the cw and pulsed excitation regimes distinguish behavior between Tb^3+^ ions at the microsecond level and this is rationalized in terms of slow energy migration and the different decay of Tb^3+^ at two different crystal environments. The quenching of emission with increasing Tb^3+^ concentration is only moderate and emission is strong even in the 90% Tb^3+^‐doped sample.

Considering the crystal structure of TbPO_4_ (Figure S2, Supporting Information), the migration between Tb^3+^ ions is expected to be 3D. Consideration of the selection rules for ^7^F_6_–^5^D_4_ migration^20^ shows that it is forbidden to first order by the exchange mechanism and although spin‐forbidden to first order, it could be due to the EQ–EQ or ED–ED mechanisms, where ED represents forced electric dipole.

### 3.3 Energy Transfer from Tb^3+^ to Eu^3+^ in the LaPO_4_ Host

#### 3.3.1 Electronic Spectra

**Figure 6:**
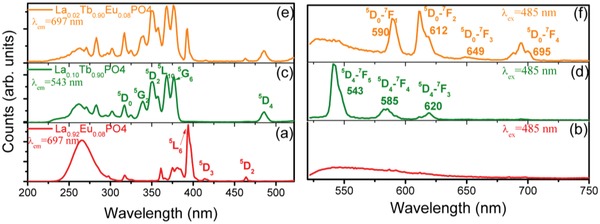
The 295 K excitation and emission spectra of a,b) La_0.92_Eu_0.08_PO_4_, c,d) La_0.10_Tb_0.90_PO_4_, and e,f) La_0.02_Tb_0.90_Eu_0.08_PO_4_ on different scales.

We have investigated the scenario of high Tb^3+^ donor concentrations in the ET from Tb^3+^ to Eu^3+^. **Figure** **6**a shows the excitation spectrum of La_0.92_Eu_0.08_PO_4_ when monitoring the Eu^3+ 5^D_0_ →^7^F_4_ emission at 697 nm. All features but one correspond to transitions from the electronic ground state to 4f^6^ excited states of Eu^3+^, with the strongest band at 393 nm corresponding to ^7^F_0_ →^5^L_6_. The broad feature with maximum at 265 nm is the Eu‐O charge transfer band. Notice that there is no Eu^3+^ absorption band near 480–500 nm so when exciting this sample with 485 nm radiation, as Figure 6b shows on an expanded ordinate scale, there are no emission bands present. The excitation spectrum of La_0.10_Tb_0.90_PO_4_, Figure 6c exhibits the ^7^F_6_ →^5^D_4_ absorption band at 485 nm, together with transitions to higher excited 4f^8^ states of Tb^3+^, and to 4f^7^5d^1^ at 260 nm. Note the absence of the broad, strong band at 316 nm observed by Chen and co‐workers.^29^ Hence, when exciting La_0.10_Tb_0.90_PO_4_ at 485 nm, Figure 6d, the emission corresponds to ^5^D_4_ →^7^F*_J_* transitions, with the strongest being with *J* = 5 at 543 nm. The excitation spectrum, Figure 6e, of the codoped sample La_0.02_Tb_0.90_Eu_0.08_PO_4_ when monitoring the Eu^3+^ emission from ^5^D_0_ exhibits features of both Eu^3+^ and Tb^3+^, thereby demonstrating ET from Tb^3+^ to Eu^3+^. Now, when exciting into the Tb^3+^ absorption band at 485 nm in La_0.02_Tb_0.90_Eu_0.08_PO_4_ (Figure 6f), the emission from Tb^3+^ is mainly quenched and all bands correspond to Eu^3+ 5^D_0_ →^7^F*_J_* transitions. The time‐resolved spectra of La_0.05_Tb_0.90_Eu_0.05_PO_4_ show that the quenching of Eu^3+^ emission (due to efficient energy transfer from Tb^3+^ to Eu^3+^) is more complete compared with La_0.75_Tb_0.20_Eu_0.05_PO_4_ (Figure S3, Supporting Information). There is no obvious emission from Tb^3+^ in La_0.05_Tb_0.90_Eu_0.05_PO_4_ even when the delay time is only 100 ns.

#### 3.3.2 Efficiency of Energy Transfer

**Figure 7:**
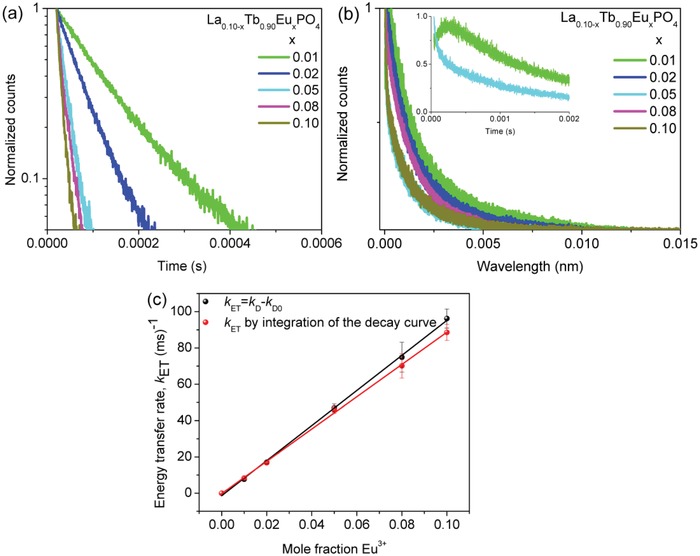
Luminescence decay curves of La_0.10−_ *_x_*Tb_0.90_Eu*_x_*PO_4_ nanocrystals: a) Tb^3+^ emission: λ_ex_ = 485 nm, λ_em_ = 543 nm) and b) Eu^3+^ emission: λ_ex_ = 485 nm, λ_em_ = 697 nm. Notice the logarithmic ordinate scale in (a). The inset in (b) shows the decay at shorter times for the samples of La_0.09_Tb_0.90_Eu_0.01_PO_4_ and La_0.05_Tb_0.90_Eu_0.05_PO_4_. c) Energy transfer rate from Tb^3+^ to Eu^3+^ in La_0.10−_ *_x_*Tb_0.90_Eu*_x_*PO_4_ as a function of mole fraction of Eu^3+^ calculated by two methods.

The efficiency of ET from Tb^3+^ to Eu^3+^ in La_0.10−_ *_x_*Tb_0.90_Eu*_x_*PO_4_ was calculated by two methods. **Figure** **7**a shows the decay curves of Tb^3+^ emission in La_0.10−_ *_x_*Tb_0.90_Eu*_x_*PO_4_ nanocrystals and the fitted monoexponential lifetime decreases from 1.6 ms (*x* = 0) with increasing of Eu^3+^ mole ratio to 0.01 ms (*x* = 0.1) (**Table** **1**, column 2). If the decay is monoexponential, the energy transfer rate from Tb^3+^ to Eu^3+^ is given by the difference between the decay rate of donor when in the presence and absence of acceptor [(1/τ_D_)−(1/τ_D0_)]. These values are listed in column 4 of Table 1 and span from 8 to 99 ms^−1^. When the decay curve is not monoexponential, the average decay time, τ_D′_, can be expressed as $\tau_{D\prime }=\int_{0}^{\infty }\frac{I(t)}{I(0)}\;dt.$ The energy transfer rate is then calculated according to column 5 of Table 1. The similarity of values in columns 4 and 5 shows that the deviation from Tb^3+^ monoexponential decay in La_0.10−_ *_x_*Tb_0.90_Eu*_x_*PO_4_ nanocrystals is relatively small. The relationship between the concentration of Eu^3+^ and the ET rate calculated by the two methods is plotted in Figure 7c. The linear relation demonstrates that the ET involves one Tb^3+^ ion and one Eu^3+^ ion and is not a three‐body process. The efficiency of ET from Tb^3+^ to Eu^3+^ achieves 99% for *x* = 0.05 (Table 1, column 6) and the ET rate is quite fast, 4.9 × 10^4^ s^−1^, accounting for the near‐monoexponential decay in Figure 7a.

**Table 1:** Summary of Tb^3+^ lifetime (τ_D_), decay rate (*k*_D_), energy transfer rate (*k*_ET_), and efficiency (η) of La_0.10−_ *_x_*Tb_0.90_Eu*_x_*PO_4_ nanocrystals (λ_ex_ = 485 nm, λ_em_ = 543 nm). For *x* = 0, *k*_D_ = *k*_D0_

*x*	τ_D_ [ms]	*k*_D_ [ms^−1^]	*k*_ET_ = *k*_D_ −*k*_D0_ [ms^−1^]	*k*_ET_ = [1/$\int_{0}^{\infty }\frac{I(t)}{I(0)}dt$]−*k*_D0_ [ms^−1^]	η%
0	1.6	0.625	0	0	–
0.01	0.11	9.09	8.47	8.90	93
0.02	0.058	17.24	16.62	16.16	96
0.05	0.020	50	49.38	48.32	99
0.08	0.015	66.67	66.04	63.16	99
0.1	0.010	100	99.38	91.86	99

Figure 7b shows the decay of ^5^D_0_ Eu^3+^ emission for various concentrations of Eu^3+^ in the codoped La_0.10−_ *_x_*Tb_0.9_Eu*_x_*PO_4_ samples. The curves have been fitted by biexponential decay functions representing a donor–acceptor process in Figure S4 (Supporting Information). The donor lifetime was set as that in column 2 of Table 1. At low Eu^3+^ concentrations, the build‐up process from population by Tb^3+ 5^D_4_ is clearly visible (refer to the inset in Figure 7b for *x* = 0.01). However, with increasing *x* the Tb^3+^ decay is faster and the build‐up is too fast to be observed in the figures (Figure S4, Supporting Information, *x* = 0.05–0.10). The fitted ^5^D_0_ Eu^3+^ lifetime does not change greatly—from 1.61 ms for *x* = 0.01 to 1.28 ms for *x* = 0.10—but the variation can be fitted by a monoexponential function (Figure S4f, Supporting Information). The depopulation of the Eu^3+ 5^D_0_ state is also due to a two‐body process (Figure S4g, Supporting Information).

### 3.4 Migration and Mechanism of Energy Transfer between Tb^3+^ and Eu^3+^

**Figure 8:**
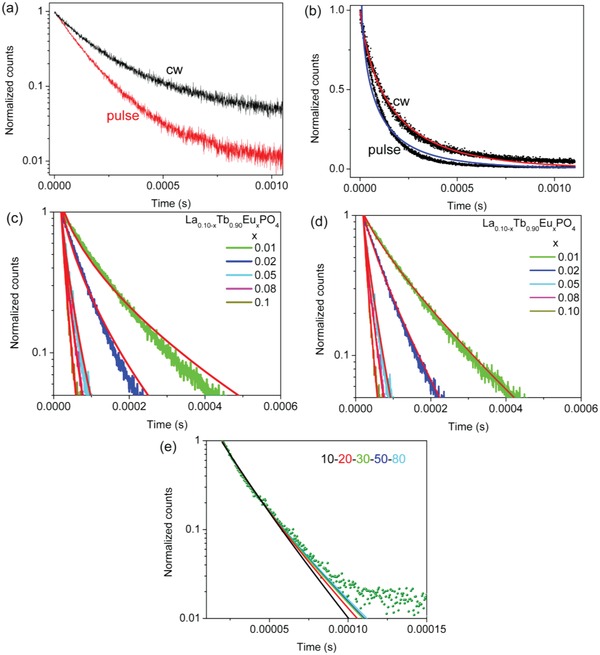
a) The decay curves of La_0.09_Tb_0.90_Eu_0.01_PO_4_ after pulsed and cw excitation. b) Fits to these curves on a linear ordinate scale using Equation (9a) for cw and Equation (3) for pulsed excitation. Fits for the series La_1−_ *_x_*Tb_0.90_Eu*_x_*PO_4_ using c) the Inokuti–Hirayama equation, Equation (3) and d) the Dornhauf–Heber equation, Equation (5). e) Sensitivity of the fits using Equation (5) to the number of shells employed, for La_0.02_Tb_0.90_Eu_0.08_PO_4_.

As discussed above for La_0.10_Tb_0.90_PO_4_, the rate of excitation migration between Tb^3+^ ions is not fast. The ^5^D_4_ Tb^3+^ emission decays after pulsed and switched‐off continuous wave excitation in the Tb^3+^,Eu^3+^ doped system are displayed on a logarithmic ordinate scale in **Figure** **8**a and show that the migration rate between Tb^3+^ ions is slower than the microsecond time scale. The fits to the curves using Equations (9a) and (3) are shown on a linear scale in Figure 8b and from the determined ratio of *c*_A_/*c*_0_, both give the critical distance *R*_0_ as 17 Å using Equation (4). Attempts to calculate the diffusion constant using the Yakota–Taminoto Equation (8), requiring one fixed and four variable parameters, were unsuccessful because there are too many parameters and the fits are not unique.

Two models were employed to investigate the ET mechanism for the entire series of La_0.10−_ *_x_*Tb_0.90_Eu*_x_*PO_4_ luminescence decays of Tb^3+^. The Inokuti–Hirayama equation (Equation (3)) gave the superior fits for *s* = 6 (Figure 8c, **Table** **2**). This ET model is based on the assumption that there is a continuous distribution of the acceptor sites around the donors. Dornauf and Heber^24^ derived the model, Equation (5), in which the “permitted” donor–acceptor separations are determined by the structure of the crystal lattice. Table S3 (Supporting Information) gives the radial distances from a donor ion to the acceptor sites in the first 80 shells in the LaPO_4_ host lattice and the number of acceptor sites in each shell. For this system, the number of discrete lattice sites around the donor was taken to be *Z* = 115. This corresponds to a sphere with radius Rk=12.4863Å. The curves can be well‐fitted with Equation (5) only when *s* = 6 which means that the multipolar mechanism is ED–ED transfer. (Figure 8d) The latter fits used two adjustable parameters, *I*_D_(0) and *R*_0_, and the values of *R*_0_ are listed in **Table** **3**. The critical transfer distances values vary over a narrow range and are slightly smaller than the values calculated in Table 2 using the model of Inokuti–Hirayama. Figure 8e shows the sensitivity of the fits using Equation (5) to the number of shells employed. The fits improve when more shells are employed.

**Table 2:** Fits of Tb^3+^ emission decay in La_0.10−_ *_x_*Tb_0.90_Eu*_x_*PO_4_ using the Inokuti–Hirayama equation, Equation (3), with τ_D_ = 0.00148 s

La_0.10−_ *_x_*Tb_0.90_Eu*_x_*PO_4_	*c*_A_ [Å^−3^]	*c*_A_/*c*_0_	*c*_0_ [Å^−3^]	*R*_0_ [Å]	*R* ^2^_adj_
0.01	0.00015	3.44	4.22 × 10^−5^	17.82	0.9898
0.02	0.00029	5.61	5.18 × 10^−5^	16.64	0.9927
0.05	0.00073	11.78	6.16 × 10^−5^	15.71	0.9801
0.08	0.00116	15.08	7.70 × 10^−5^	14.58	0.9863
0.10	0.00145	20.04	7.24 × 10^−5^	14.88	0.9363

**Table 3:** Fits of emission decay of Tb^3+^ in La_0.10−_ *_x_*Tb_0.90_Eu*_x_*PO_4_ using the Dornauf–Heber equation (Equation (5)): τ_D_= 0.00148 s, *Z* = 115 discrete lattice sites arranged in 80 shells

La_0.10−_ *_x_*Tb_0.90_Eu*_x_*PO_4_	*c*_A_ [Å^−3^]	*s*	*R*_0_ [Å]	$R_{\mathrm{adj}}^{2}$
0.01	0.00015	6	14.61	0.9931
0.02	0.00029	6	14.31	0.9952
0.05	0.00073	6	14.08	0.9788
0.08	0.00116	6	13.32	0.9894
0.10	0.00145	6	13.63	0.9345

### 3.5 Color Variation on Doping LaPO_4_ with Tb^3+^ and Eu^3+^

**Figure 9:**
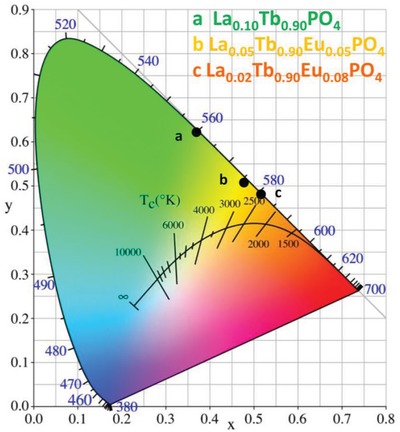
CIE chromaticity diagram for the La_0.10_Tb_0.90_PO_4_, La_0.05_Tb_0.90_Eu_0.05_PO_4_, and La_0.02_Tb_0.90_Eu_0.08_PO_4_ nanocrystals.

**Figure** **9** shows the CIE chromaticity coordinates (*x*, *y*) for three samples of the doped LaPO_4_ nanocrystals. The singly Tb^3+^‐doped materials exhibit green emission (*x* = 0.37, *y* = 0.62) but the color changes to orange for the doubly doped nanocrystals: (*x* = 0.48, *y* = 0.51) for La_0.05_Tb_0.90_Eu_0.05_PO_4_ and (*x* = 0.52, *y* = 0.48) for La_0.02_Tb_0.90_Eu_0.08_PO_4_.

## 4 Conclusions

Luminescence quenching by migration of the excitation energy throughout the lattice which terminates at trap sites often limits the concentration of doped lanthanide ions in a solid‐state material to less than a few mol%. The energy migration in highly doped materials has been stated as very fast or slow but no related experiment has proven the suggestion. We have chosen a system with a high mole ratio of terbium in order to investigate the migration process. We focused upon the luminescence performance of Tb^3+^ doped into the LaPO_4_ host lattice by employing pulsed and switched‐off continuous wave excitation. The experiments gave different Tb^3+^ luminescence decay profiles so that a fast migration regime does not occur following excitation. In fact, the luminescence lifetime is only reduced by ≈50% on going from a very dilute sample to La_0.10_Tb_0.90_PO_4_. The quenching has been associated with the presence of interstitial water. The process of migration itself is an ET process between like ions so that it depends upon the oscillator strengths of the donor and acceptor transitions and the spectral overlap integral. The slow migration between Tb^3+^ ions results from (i) the weak oscillator strength of the ^5^D_4_–^7^F_6_ transitions (taken as 3 × 10^−7^ 50, 51) and (ii) the diminished spectral overlap between absorption and emission because the spread of the ^7^F_6_ *J*‐multiplet is larger than *kT* at room temperature. Figure S1 (Supporting Information) shows that the migration rate, *k*_Df_, is similar to the radiative rate. The value herein is similar to the migration rate of 339 s^−1^ found in Sr_3_Tb_0.90_Eu_0.10_(PO_4_)_3_ 50 where the Tb—Tb distance is also ≈4 Å.

The mechanism of ET between Tb^3+^ and Eu^3+^ has been previously studied with conflicting results, which claimed that ED–ED,^13^, ^14^, ^15^, ^16^ ED–EQ,^17^ or exchange^18^, ^19^ is the dominant interaction mechanism. Naturally, the mechanism may depend upon the crystal structure, determining the site symmetries of these ions and the distance between them. In the present study, the energy transfer mechanism between Tb^3+^ and Eu^3+^ has been studied systematically by different models and found to be ED–ED, where ED represents the forced electric dipole mechanism.

The efficiency of ET from Tb^3+^ to Eu^3+^ approaches 100% even for lightly doped Eu^3+^ samples (Table 1). The ET rate is ≈10^5^ s^−1^ for Tb_0.90_Eu_0.10_PO_4_, and (from Table 3) the donor–acceptor ET parameter $\alpha_{\mathrm{DA}}^{\left(6\right)}$ is calculated to be 4.3 × 10^−39^ cm^6^ s^−1^. This value is considerably greater than those for Y_2_O_3_:Tb^3+^,Eu^3+^ (2.0 × 10^−41^ cm^6^ s^−1^)^52^ and Sr_3_Tb_0.90_Eu_0.10_(PO_4_)_3_ (4.8 × 10^−41^ cm^6^ s^−1^).^50^ The luminescence decay of Tb^3+^ can be well‐fitted by a model, which neglects diffusion and follows the ED–ED mechanism. The very efficient ET between Tb^3+^ and Eu^3+^ was previously reported for hexagonal hydrated TbPO_4_:Eu^3+^ nanocrystals.^[29]^ We also concur with this study that diffusion does not play a major role in this ET process, but we do not observe fast energy migration in singly Tb^3+^‐doped LaPO_4_. In future, we will examine the ET between Tb^3+^ and Eu^3+^ in the regime of low Tb^3+^ concentrations.

## 5 Experimental Section

*Synthesis of LaPO_4_:Tb^3+^, Eu^3+^ Nanomaterials*: The materials employed and their purity are listed in the Supporting Information. La_1−_ *_x_*Tb*_x_*PO_4_ (*x* = 0.001, 0.05, 0.10, 0.20, 0.30, 0.50, 0.70, 0.90) and La_0.10−_ *_y_*Tb_0.90_Eu*_y_*PO_4_ (*y* = 0.01, 0.02, 0.05, 0.08,0.10) nanoparticles were prepared by a modified method of Haase and co‐workers.^[30,31]^ A clear solution of 1 mmol lanthanide chlorides (LaCl_3_·7H_2_O, TbCl_3_·6H_2_O and EuCl_3_·7H_2_O) in 1 mL ethylene glycol was mixed with 1 mmol triethyl phosphate. Then 10 mL of ethylene glycol (99%, Dieckman) was added and the water released by the hydrated salts was removed under vacuum at 105 °C. The mixture was purged with nitrogen in a Schlenkline. At a temperature below 50 °C, 3 mmol trioctylamine (TOA) was added, followed by 1.4 mmol of a solution of phosphoric acid in ethylene glycol. The reaction mixture was kept for 6 h under nitrogen at 180 °C. After cooling, the nanocrystals were precipitated from the reaction mixture by addition of methanol, washed with methanol two times, and dried under vacuum.

*Instrumental Methods*: X‐ray diffraction patterns of crystals were recorded with a Bruker AXS D8 Advance X‐Ray Diffractometer equipped with a non‐monochromated Cu Kα X‐ray source (λ = 1.5418 Å). TEM and HRTEM images were obtained by a Tecnai G2 20 S‐TWIN Transmission Electron Microscope. Infrared spectra were recorded at room temperature in the range 400–4000 cm^−1^ using a Perkin Elmer Paragon 1000 PC spectrometer with resolution of 4 cm^−1^. The electronic emission and excitation spectra were recorded by a Horiba Fluorolog‐3 spectrophotometer using a 450 W continuous xenon lamp as the light source and the signal was detected by a Hamamatsu R928 photomultiplier. Luminescence lifetimes were measured by a digital phosphor oscilloscope (DSO9104A Oscilloscope from Agilent Technologies: 1 GHz, 20 GSa s^−1^) with a Nd:YAG pulsed laser as excitation source, a third‐order harmonic generator (120 mJ), and an optical parameter oscillator (OPO, Spectra‐Physics versaScan and UVScan: pulse duration 8 ns, repetition frequency 10 Hz). Continuous wave excitation was provided by a 488 nm laser power (MDL‐III‐488L‐20 mW) with a shut‐off time of 25 µs (Figure S5, Supporting Information).

## Conflict of Interest

The authors declare no conflict of interest.

## Supporting information

Supplementary

## References

[1] L. Zhou , W. Zhou , F. Pan , R. Shi , L. Huang , H. Liang , P. A. Tanner , X. Du , Y. Huang , Y. Tao , L. Zheng , Chem. Mater. 2016, 28, 2834.

[2] K. Kompe , H. Borchert , J. Storz , A. Lobo , S. Adam , T. Moller , M. Haase , Angew. Chem., Int. Ed. 2003, 42, 5513. 10.1002/anie.200351943 14618592

[3] M. Back , R. Marin , M. Franceschin , N. S. Hancha , F. Enrichi , E. Travea , S. Polizzi , J. Mater. Chem. C 2016, 4, 1906.

[4] J. T. Van Wijngaarden , S. Scheidelaar , T. J. H. Vlugt , M. F. Reid , A. Meijerink , Phys. Rev. B 2010, 81, 15112.

[5] J. Pisarska , A. Kos , W. A. Pisarski , Spectrochim. Acta, Part A 2014, 129, 649. 10.1016/j.saa.2014.04.142 24824577

[6] H. Lin , E. Y. B. Pun , X. Wang , X. Liu , J. Alloys Compd. 2005, 390, 197.

[7] Z. Wang , P. Li , Z. Yang , Q. Guo , J. Lumin. 2014, 151, 170.

[8] X. Min , Z. Huang , M. Fang , Y. Liu , C. Tang , X. Wu , Inorg. Chem. 2014, 53, 6060. 24884208 10.1021/ic500412r

[9] M. Xing , W. Cao , H. Zhong , Y. Zhang , X. Luo , Y. Fu , W. Feng , T. Pang , X. Yang , J. Alloys Compd. 2011, 509, 5725.

[10] L. Guo , Y. Wang , J. Zhang , Y. Wang , P. Dong , Nanoscale Res. Lett. 2012, 7, 636. 23171624 10.1186/1556-276X-7-636 PMC3533901

[11] J. D. L. Dutra , N. B. D. Lima , R. O. Freire , A. M. Simas , Sci. Rep. 2015, 5, 13695. 26329420 10.1038/srep13695 PMC4557129

[12] D. M. Moran , P. S. May , F. S. Richardson , Chem. Phys. 1994, 186, 77.

[13] Y. Liu , G. Qian , Z. Wang , M. Wang , Appl. Phys. Lett. 2005, 86, 071907.

[14] X. Wang , X. Wang , X. Zheng , L. Zhang , J. Alloys Compd. 2015, 632, 269.

[15] M. Runowski , A. Shyichuk , A. Tymiński , T. Grzyb , V. Lavín , S. Lis , ACS Appl. Mater. Interfaces 2018, 10, 17269. 29722259 10.1021/acsami.8b02853

[16] M. O. Rodrigues , J. D. L. Dutra , L. A. O. Nunes , G. F. de Sá , W. M. de Azevedo , P. Silva , F. A. A. Paz , R. O. Freire , S. A. Júnior , J. Phys. Chem. C 2012, 116, 19951.

[17] M. T. Berry , P. S. May , Q. Hu , J. Lumin. 1997, 71, 269.

[18] K. Sawada , T. Nakamura , S. Adachi , J. Alloys Compd. 2016, 678, 448.

[19] L. Wang , Z. Yang , Y. Li , R. Yang , Z. Dai , S. Hu , L. Sun , Y. Tong , Spectrochim. Acta, Part A 2018, 202, 76. 10.1016/j.saa.2018.05.015 29778708

[20] P. A. Tanner , L. Zhou , C. Duan , K.‐L. Wong , Chem. Soc. Rev. 2018, 47, 5234. 29938282 10.1039/c8cs00002f

[21] I. Carrasco , F. Piccinelli , I. Romet , V. Nagirnyi , M. Bettinelli , J. Phys. Chem. C 2018, 122, 6858.

[22] I. Carrasco , F. Piccinelli , M. Bettinelli , J. Phys. Chem. C 2017, 121, 16943.

[23] G. Blasse , Prog. Solid State Chem. 1988, 18, 79.

[24] H. Dornauf , J. Heber , J. Lumin. 1980, 22, 1.

[25] F. K. Fong , D. J. Diestler , J. Chem. Phys. 1972, 56, 2875.

[26] V. Lupei , J. Lumin. 1991, 48, 157.

[27] M. Bettinelli , C. D. Flint , J. Phys.: Condens. Matter 1990, 2, 8417.

[28] M. Tachihante , A. Arbus , M. T. Fournier , J. C. Cousseins , J. Less Common Met. 1985, 112, 83.

[29] Y. Ruan , Q. Xiao , W. Luo , R. Li , X. Chen , Nanotechnology 2011, 22, 275701. 21597160 10.1088/0957-4484/22/27/275701

[30] R. Komban , K. Koempe , M. Haase , Cryst. Growth Des. 2011, 11, 1033.

[31] K. Riwotzki , H. Meyssamy , A. Kornowski , M. Haase , J. Phys. Chem. B 2000, 104, 2824.

[32] W. M. Yen , R. M. Selzer , Laser Spectroscopy of Solids, 2nd ed., Springer‐Verlag, Berlin 1986, Ch. 3.

[33] D. L. Huber , Phys. Rev. B 1979, 20, 2307.

[34] M. Inokuti , F. Hirayama , J. Chem. Phys. 1965, 43, 1978.

[35] F. T. Rabouw , S. A. den Hartog , T. Senden , A. Meijerink , Nat. Commun. 2014, 5, 3610. 24694758 10.1038/ncomms4610

[36] D. Yu , F. T. Rabouw , W. Q. Boon , T. Kieboom , S. Ye , Q. Zhang , A. Meijerink , Phys. Rev. B 2014, 90, 165126.

[37] M. Yokota , O. Taminoto , J. Phys. Soc. Jpn. 1967, 22, 779.

[38] I. R. Martín , V. D. Rodríguez , U. R. Rodríguez‐Mendoza , V. Lavín , E. Montoya , D. Jaque , J. Chem. Phys. 1999, 111, 1191.

[39] A. I. Burshtein , Sov. Phys. ‐ JETP 1972, 35, 882.

[40] K. B. Eisenthal , S. Siegel , J. Chem. Phys. 1964, 41, 652.

[41] H. Siebold , J. Heber , J. Lumin. 1981, 22, 297.

[42] D. F. Mullica , D. A. Grossie , L. A. Boatner , Inorg. Chim. Acta 1985, 109, 105.

[43] W. O. Milligan , D. F. Mullica , G. W. Beall , L. A. Boatner , Inorg. Chim. Acta 1983, 70, 133.

[44] K. Nakamoto , Infrared and Raman Spectra of Inorganic and Coordination Compounds, *Part A*, 5th ed, Wiley, New York 1997.

[45] W. Di , X. Wang , P. Zhu , B. Chen , J. Solid State Chem. 2007, 180, 467.

[46] J. J. H. A. van Hest , G. A. Blab , H. C. Gerritsen , C. de Mello Donega , A. Meijerink , J. Phys. Chem. C 2017, 121, 19373. 10.1021/acs.jpcc.7b06549 PMC5592647 28919934

[47] N. J. J. Johnson , S. He , S. Diao , E. M. Chan , H. Dai , A. Almutairi , J. Am. Chem. Soc. 2017, 139, 3275. 28169535 10.1021/jacs.7b00223

[48] M. Chua , P. A. Tanner , J. Lumin. 1995, 66, 203.

[49] J. Dexpert‐Ghys , R. Mauricot , M. D. Faucher , J. Lumin. 1996, 69, 203.

[50] M. Bettinelli , F. Piccinelli , A. Speghini , J. Ueda , S. Tanabe , J. Lumin. 2012, 132, 27.

[51] Z. Yahiaoui , M. A. Hassairi , M. Dammak , E. Cavalli , F. Mezzadri , J. Lumin. 2018, 194, 96.

[52] T. K. Anh , T. Ngoc , P. T. Nga , P. Long , W. Strek , J. Lumin. 1988, 39, 215.

