# Supplementary material for: Energy Transfer between Tb3+ and Eu3+ in LaPO4: Pulsed versus Switched‐off Continuous Wave Excitation
Source: Adv Sci (Weinh). 2019 Apr 5;6(10):1900487. doi: 10.1002/advs.201900487 (PMC6524098; doi:10.1002/advs.201900487)
Supplement: Supplementary file 1 — Supplementary [file ADVS-6-1900487-s001.pdf]

Copyright WILEY-VCH Verlag GmbH & Co. KGaA, 69469 Weinheim, Germany, 2019.

ADVANCED SCIENCE
Open Access

Supporting Information

for *Adv. Sci.,* DOI: 10.1002/advs.201900487

Energy Transfer between Tb^{3+} and Eu^{3+} in $LaPO_4$: Pulsed versus Switched-off Continuous Wave Excitation

Yuxia Luo, Zhenyu Liu, Hon Tung Wong, Lei Zhou, Ka-Leung Wong, Kwok Keung Shiu, and Peter A. Tanner**

Copyright WILEY-VCH Verlag GmbH & Co. KGaA, 69469 Weinheim, Germany, 2019.

Supporting Information

Energy Transfer between Tb^{3+} and Eu^{3+} in $LaPO_4$: pulsed *vs* switched-off continuous wave excitation

Yuxia Luo, Zhenyu Liu, Hon Tung Wong, Lei Zhou, Ka-Leung Wong*, Kwok Keung Shiu, Peter A. Tanner*

Department of Chemistry, Hong Kong Baptist University, 224 Waterloo Road, Kowloon, Hong Kong, S.A.R., P. R. China
*Corresponding authors
E-mail: peter.a.tanner@gmail.com (P.A.T.)
E-mail: klwong@hkbu.edu.hk (K.-L.W.)

Table S1. Fitted Tb^{3+} emission decay by monoexponential decay rate of $La_{1-x}Tb_xPO_4$ nanocrystals (λ_{ex} = 485 nm, λ_{em} = 543 nm) 2

Figure. S1. Plot of k(long)–k(rad) = k_{Df} *vs* Tb^{3+} molar concentration in $TbPO_4$.....................2

Table S2. Fitted Tb^{3+} emission decay by biexponential decay rate of $La_{1-x}Tb_xPO_4$ nanocrystals (λ_{ex} = 485 nm, λ_{em} = 543 nm) 2

Figure S2. The crystal structure of $TbPO_4$ (left) and the distances from other Tb ions to the marked one....................3

Figure S3. The time resolved spectra of $La_{1.0-x}Tb_{0.90}Eu_xPO_4$ excited by 485 nm. 3

Figure S4. (a)-(e) Biexponential fits for decay of Eu^{3+} emission in $La_{1.00-x}Tb_{0.90}Eu_xPO_4$. (f) Plot of fitted Eu^{3+} lifetime against mole fraction of Eu^{3+} in $La_{1.00-x}Tb_{0.90}Eu_xPO_4$ and fitting by monoexponential decay. (g) Plot of Eu^{3+} energy transfer rate against mole fraction of Eu^{3+}. ...4

Table S3: The radial distances from a donor ion to the first 80 shells of acceptor sites and the number of acceptor sites in each shell. 4

Figure. S5. Switch-off time for laser diode....................6

Table S4. Summary of studies concerning the energy transfer between Tb^{3+} and Eu^{3+} in different systems 7

Materials employed

All of the used reagents were of analytical quality except rare earth oxides which were of spectral purity. The following reagents were used for the synthesis: lanthanide chlorides ($LaCl_3·7H_2O$, 99.999%, Sigma-Aldrich, $TbCl_3·6H_2O$, 99.9%, Sigma-Aldrich, $EuCl_3·7H_2O$, 96.0%, TCI), ethylene glycol (99%, Dieckman), triethyl phosphate, trioctylamine (TOA, 98%, Sigma-Aldrich), phosphoric acid (99.999%, Sigma-Aldrich).

Table S1. Fitted Tb^{3+} emission decay by monoexponential decay rate of $La_{1-x}Tb_xPO_4$ nanocrystals (λ_{ex} = 485 nm, λ_{em} = 543 nm)

$x Tb^{3+}$	R^2_{adj}	Lifetime [ms]	Decay rate $[ms]^{-1}$
0.001	0.99877	3.04	0.329
0.05	0.99836	2.82	0.355
0.10	0.99909	2.84	0.352
0.20	0.99970	2.74	0.365
0.30	0.99839	1.98	0.505
0.50	0.99916	1.76	0.568
0.70	0.99895	1.49	0.671
0.90	0.99627	1.48	0.676

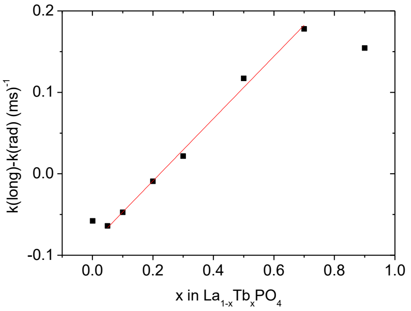

Figure S1. Plot of k(long)–k(rad) = k_{Df} *vs* Tb^{3+} molar concentration in $TbPO_4$. k(long) was measured from the decay curve between 10-20 ms after the excitation pulse whereas k(rad) was taken as 1/(3.04 ms).

Table S2. Fitted Tb^{3+} emission decay by biexponential decay rate of $La_{1-x}Tb_xPO_4$ nanocrystals (λ_{ex} = 485 nm, λ_{em} = 543 nm)

$x Tb^{3+}$	R^2_{adj}	A_1	τ_1 [ms]	A_2	τ_2 [ms]
0.001	0.99921	0.233	1.16	1.029	3.31
0.05	0.99935	0.551	1.62	0.724	3.60
0.10	0.99964	0.327	1.30	0.956	3.23
0.20	0.99986	0.176	1.16	1.111	2.91
0.30	0.99982	0.601	0.92	0.874	2.43
0.50	0.99992	0.474	0.70	1.093	1.99
0.70	0.99991	0.615	0.57	1.134	1.70
0.90	0.99988	1.314	0.39	1.030	1.78

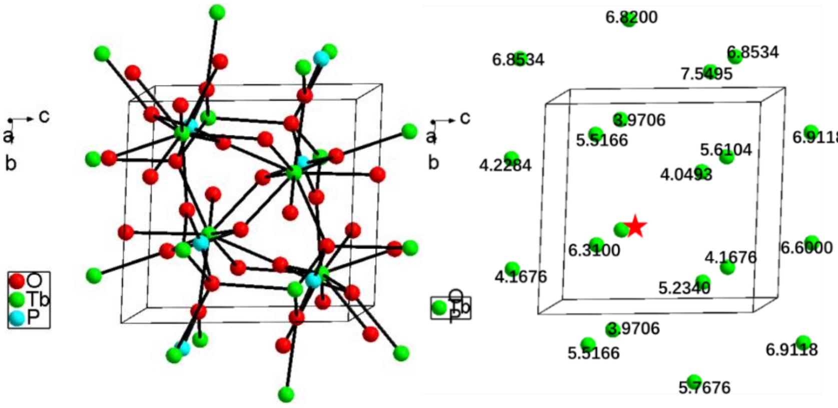

Figure S2. The crystal structure of $TbPO_4$ (left) and the distances from other Tb ions to the marked one.

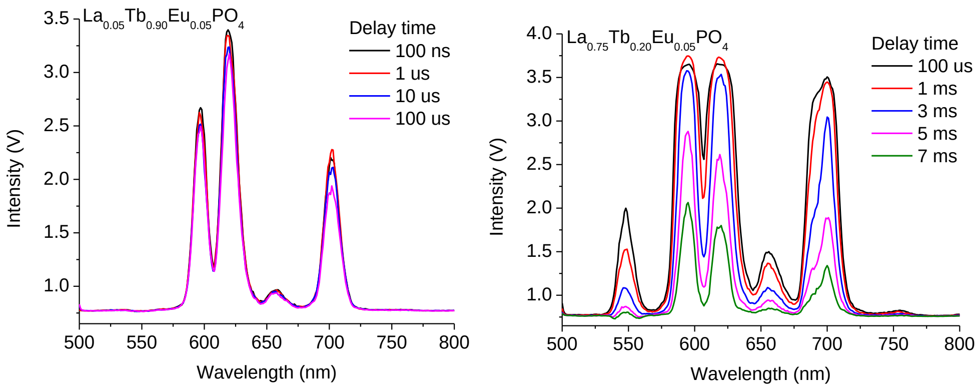

Figure S3. The time resolved spectra of $La_{1.0\text{-}x}Tb_{0.90}Eu_xPO_4$ excited by 485 nm.

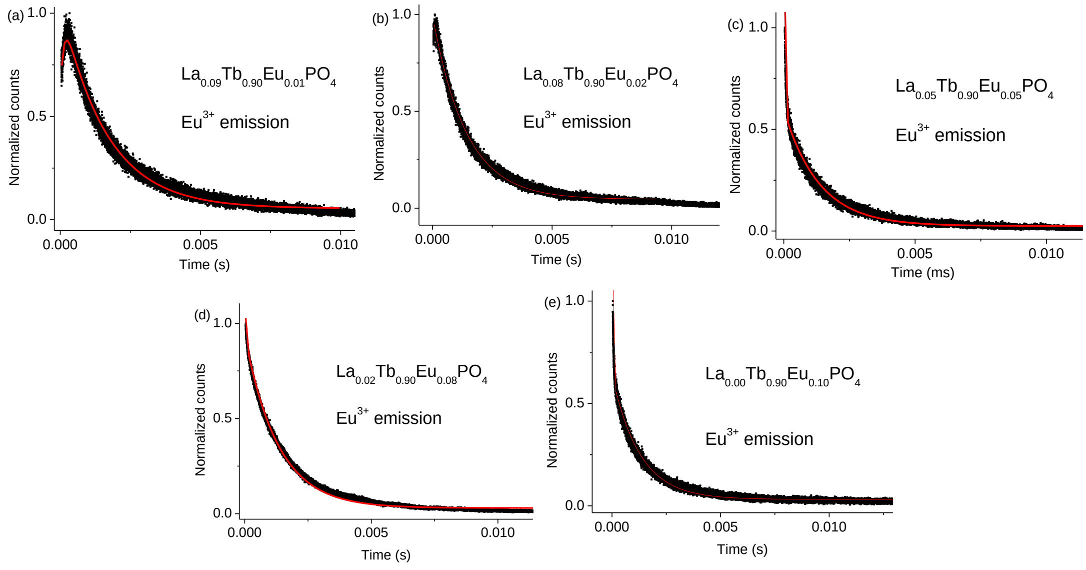

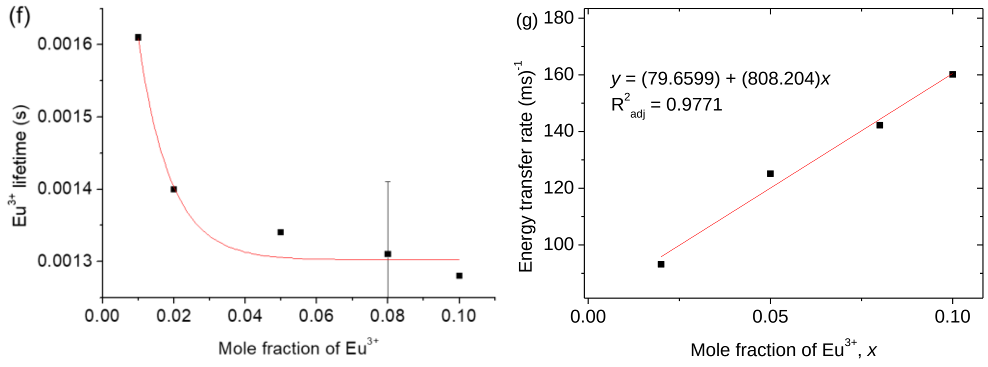

Figure S4. (a)-(e) Biexponential fits for decay of Eu^{3+} emission in $La_{1.00-x}Tb_{0.90}Eu_xPO_4$. (f) Plot of fitted Eu^{3+} lifetime against mole fraction of Eu^{3+} in $La_{1.00-x}Tb_{0.90}Eu_xPO_4$ and fitting by monoexponential decay. (g) Plot of Eu^{3+} energy transfer rate against mole fraction of Eu^{3+}.

Table S3. The radial distances from a donor ion to the first 80 shells of acceptor sites and the number of acceptor sites in each shell.

Shell	Coordination number	Distance [Å]
1	2x	3.9708
2	1x	4.0436
3	2x	4.1675
4	1x	4.2287
5	2x	5.2339
6	2x	5.5162
7	1x	5.6107
8	1x	5.7671
9	1x	5.8984
10	2x	6.31
11	2x	6.6001
12	2x	6.82
13	2x	6.8536
14	2x	6.9116
15	1x	6.9562
16	1x	7.2844
17	2x	7.5497
18	1x	7.8952
19	2x	7.9577
20	2x	8.0928
21	1x	8.3649
22	2x	8.4193

23	1x	8.9018
24	2x	8.9854
25	2x	8.9883
26	1x	8.9919
27	2x	9.082
28	2x	9.2911
29	2x	9.2915
30	2x	9.326
31	2x	9.4905
32	2x	9.4908
33	2x	9.5297
34	1x	9.616
35	2x	9.6276
36	1x	9.6952
37	1x	9.7189
38	1x	9.7195
39	1x	9.8498
40	1x	9.8875
41	1x	10.0966
42	1x	10.1698
43	1x	10.1701
44	1x	10.3215
45	1x	10.3728
46	2x	10.4303
47	2x	10.48
48	2x	10.4806
49	1x	10.6737
50	1x	10.7968
51	1x	10.7975
52	1x	10.8188
53	1x	10.8193
54	1x	10.9018
55	1x	11.1105
56	2x	11.1109
57	1x	11.1383
58	1x	11.1388
59	2x	11.1597
60	2x	11.1679
61	1x	11.2357
62	1x	11.3657
63	1x	11.4426
64	1x	11.7665

65	2x	11.821
66	2x	11.8657
67	1x	11.9645
68	1x	11.9663
69	1x	12.0003
70	1x	12.0011
71	1x	12.0301
72	1x	12.1099
73	1x	12.1104
74	1x	12.1591
75	2x	12.2449
76	2x	12.2451
77	1x	12.2633
78	1x	12.2639
79	2x	12.4154
80	2x	12.4863
81	1x	12.5288

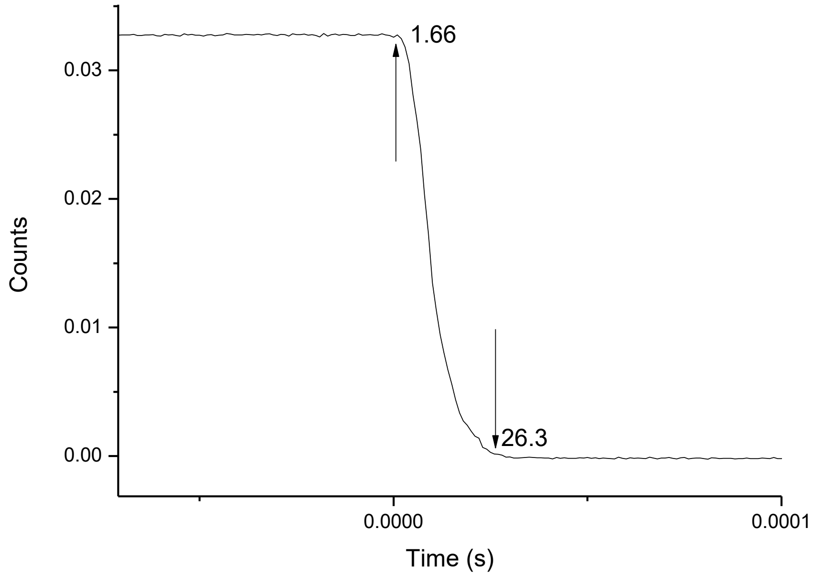

Figure S5. Switch-off time for laser diode. The times are marked in microseconds so that the switch-off time is ~25 μs.

Table S4. Summary of studies concerning the energy transfer between Tb^{3+} and Eu^{3+} in different systems

System	Preparation method	Conc.%	T (K)	Closest distance (Å)	Cation C.N.	λ_{exc} (nm)	η(ET) %	ET rate $(ms)^{-1}$	ET mechanism	QE %	Ref.
$LaPO_4:Tb^{3+}/Eu^{3+}$	co-precipitation method	Tb 10<br>Eu 4	298			350					1
$TbPO_4:Eu^{3+}$	high temperature solid-state reactions	Eu 2.5	298	3.79		160<br>377	60		exchange interaction<br>migration-assisted		2
$YPO_4:Tb^{3+}/Eu^{3+}$	high temperature solid-state reactions	Tb 5<br>Eu 8	298	7.0			31		$^5D_4(Tb)+^7F_0(Eu)+\Delta E_{ph}$ - $^7F_4(Tb)+^5D_0(Eu)$<br>$^5D_4(Tb)+^7F_0(Eu)$ - $^7F_4(Tb)+^5D_1(Eu)+\Delta E_{ph}$		3
$LaPO_4\cdot nH_2O:Tb^{3+}/Eu^{3+}$<br>$LaPO_4:Tb^{3+}/Eu^{3+}$	microwave-assisted hydrothermal synthesis	1	298			345					4
$K_2Ln(PO_4)(WO_4):Tb^{3+},Eu^{3+}$	high temperature solid-state reactions	40-50	298		8	378		0.36		76.45 (internal,) 35.2 (external) λ_{exc}=394 nm)	5
$Zn_8[(BO_3)_3O_2(OH)_3]$: xEu^{3+}/yTb^{3+}	hydrothermal	x=5; y=0.5, 1, 1.5 and 2	298			245<br>394	11.4			3.31 λ_{exc}=254 nm	6
$Y_2O_3:Tb^{3+},Eu^{3+}$	template method	Tb 3<br>Eu 0.6	298			254<br>282<br>303					7
$ZnO:Tb^{3+},Eu^{3+}$	magnetron co-sputtering	Tb 1,2,3,4,5<br>Eu 2									8
$(Ga_{1-x-y}Tb_xEu_y)_2O_3$	metal organic decomposition (MOD)	10	20-450			266 355<br>488	60		$^5D_4(Tb)+^7F_0(Eu)$- $^7F_{6,5,4}(Tb)+^5D_{1,0}(Eu)\pm\Delta E_{JP}$<br>exchange interaction		9

$Tb_2(WO_4)_3$:Eu^{3+}	sol–gel process and electrospinning	Eu 0.3–5	298			490				10
$SrTiO_3$:Tb^{3+},Eu^{3+}	sorption	Tb 10^{-2} Eu 10^{-4}-1	298	3-5		487			Inokuti–Hirayama model	11
$NaLa(MoO_4)_2$: Tb^{3+}, Eu^{3+},	hydrothermal	total 5	298			236				12
72PbO–$18B_2O_3$–$8Al_2O_3$–$0.5Tb_2O_3$–$1.5Eu_2O_3$	state fusion method		298			378	18			13
$KBaY(MoO_4)_3$	high temperature solid-state reactions	Tb 40 Eu 1-16	298	10.1		376	26.6		dipole–dipole interaction	14
CaF_2: Tb^{3+}, Eu^{3+},	co-precipitation method	Tb 2.5 Eu 1, 2.5, 5	298			355	90		dipole–dipole interaction $I_{D0}/I_D \alpha (c_A)^{s/3}$	15
CaF_2: Eu^{2+}, Tb^{3+}	high temperature solid-state reactions	5	1523			398	64.9		exchange interaction	16
$CaCO_3$: Tb^{3+}, Eu^{3+}	carbonation method	Tb^{3+} 0.5-2.5 Eu^{3+} 2				368	66.07		$I_{D0}/I_D \alpha (c_A)^{s/3}$	17
$\{Tb_xEu_{1-x}(\alpha$- furoate$)_3(H_2O)_3\}_n$	exchange reactions		298			280 380	64-79			18
cycTb-phEu			298	10.6	8	355	81	7.1		26
			10			355	35	0.84		
$[Tb_{0.99}Eu_{0.01}(hfa)_3(dpbp)]_n$			200	I		355		1		19
$[Tb_{0.99}Eu_{0.01}(hfa)_3(dpbp)]_n$			300			355	38			19
$Y_{0.46}Ga_{1.5}Al_{1.5}(BO_3)_4$:$Tb_{0.50}Eu_{0.04}$			300	7.1	6	374	38	0.32		20
$Y_{0.34}Ga_{1.5}Al_{1.5}(BO_3)_4$:$Tb_{0.50}Eu_{0.16}$			300		6	374	74	1.50		20
$Cs_2NaTb_{0.99}Eu_{0.01}Cl_6$			293	7.6	6	488	9	0.015		21
			80			488	5	0.006		21
$Cs_2NaTb_{0.85}Eu_{0.15}Cl_6$			293	7.6	6	488	87	1.01		21
			80			488	77	0.35		21
$([(Eu_{0.5}Tb_{0.5})(C_6H_8O_4)_3$- $(H_2O)_2]$ $(C_{10}H_8N_2)$			295	4.053	9	285	45	0.59		22

$Na_3[Tb_{0.01}Eu_{0.99}(oxy)_3].2NaC1O_4.6H_2O$	298	9.4	9	488	23	0.11		[23]
	77			488	0.4	0.0017		[23]
$Sr_3Tb_{0.90}Eu_{0.10}(PO_4)_3$	298	4	6	355	93	1.67		[24]
$Ca_3Tb_{1.90}Eu_{0.10}Si_3O_{12}$	300	Est 7.94			0.94	94		[25]

1. Dong, W.; Zhang, X.; Shi, H.; Wang, N.; Zhang, W.; Li, L.; Xue, Q., Preparation and luminescence properties of color-tunable single-phased $LaPO_4:Eu^{3+}/Tb^{3+}$ phosphors. *Chemical Research in Chinese Universities* **2016,** *32* (2), 248-252.
2. Carrasco, I.; Piccinelli, F.; Romet, I.; Nagirnyi, V.; Bettinelli, M., Competition between energy transfer and energy migration processes in neat and Eu^{3+}-doped $TbPO_4$. *The Journal of Physical Chemistry C* **2018,** *122* (12), 6858-6864.
3. Yahiaoui, Z.; Hassairi, M. A.; Dammak, M.; Cavalli, E.; Mezzadri, F., Tunable luminescence and energy transfer properties in $YPO_4:Tb^{3+}$, Eu^{3+}/Tb^{3+} phosphors. *Journal of Luminescence* **2018,** *194*, 96-101.
4. Colomer, M. T.; Zur, L.; Ferrari, M.; Ortiz, A. L., Structural-microstructural characterization and optical properties of Eu^{3+},Tb^{3+}-codoped $LaPO_4{\cdot}nH_2O$ and $LaPO_4$ nanorods hydrothermally synthesized with microwaves. *Ceramics International* **2018,** *44* (11), 11993-12001.
5. Wen, D.; Feng, J.; Li, J.; Shi, J.; Wu, M.; Su, Q., $K_2Ln(PO_4)(WO_4):Tb^{3+},Eu^{3+}$ (Ln = Y, Gd and Lu) phosphors: highly efficient pure red and tuneable emission for white light-emitting diodes. *Journal of Materials Chemistry C* **2015,** *3* (9), 2107-2114.
6. Liang, P.; Liu, J. W.; Liu, Z. H., Controllable hydrothermal synthesis of $Eu^{3+}/Tb^{3+}/Dy^{3+}$ activated $Zn_8[(BO_3)_3O_2(OH)_3]$ micro/nanostructured phosphors: energy transfer and tunable emissions. *RSC Adv.* **2016,** *6* (92), 89113-89123.
7. Jiu, H.; Fu, Y.; Zhang, L.; Sun, Y.; Wang, Y., Effect of Eu, Tb codoping on the luminescent properties of Y_2O_3 hollow microspheres. *Optical Materials* **2012,** *35* (2), 141-145.
8. Huang, M.; Wang, S.; Wan, G.; Zhang, X.; Zhang, Y.; Ou, K.; Yi, L., Effect of co-doped Tb^{3+} ions on electroluminescence of $ZnO:Eu^{3+}$ LED. *Journal of Materials Science: Materials in Electronics* **2018,** *29* (9), 7213-7219.
9. Sawada, K.; Nakamura, T.; Adachi, S., Abnormal photoluminescence phenomena in (Tb^{3+}, Eu^{3+}) codoped Ga_2O_3 phosphor. *Journal of Alloys and Compounds* **2016,** *678*, 448-455.
10. Hou, Z.; Cheng, Z.; Li, G.; Wang, W.; Peng, C.; Li, C.; Ma, P.; Yang, D.; Kang, X.; Lin, J., Electrospinning-derived $Tb_2(WO_4)_3:Eu(^{3+})$ nanowires: energy transfer and tunable luminescence properties. *Nanoscale* **2011,** *3* (4), 1568-74.
11. García-Rosales, G.; Mercier-Bion, F.; Drot, R.; Lagarde, G.; Roques, J.; Simoni, E., Energy transfer from Tb^{3+} to Eu^{3+} ions sorbed on $SrTiO_3$ surface. *Journal of Luminescence* **2012,** *132* (5), 1299-1306.
12. Li, G.; Li, L.; Li, M.; Bao, W.; Song, Y.; Gan, S.; Zou, H.; Xu, X., Hydrothermal synthesis and luminescent properties of

$NaLa(MoO_4)_2:Eu^{3+},Tb^{3+}$ phosphors. *Journal of Alloys and Compounds* **2013,** *550*, 1-8.
13. Pisarska, J.; Kos, A.; Pisarski, W. A., Spectroscopy and energy transfer in lead borate glasses doubly doped with Dy^{3+}-Tb^{3+} and Tb^{3+}-Eu^{3+} ions. *Spectrochimica Acta Part A: Molecular and Biomolecular Spectroscopy* **2014,** *129*, 649-53.
14. Li, K.; Van Deun, R., Photoluminescence and energy transfer properties of a novel molybdate $KBaY(MoO_4)_3:Ln^{3+}$ ($Ln^{3+} = Tb^{3+}$, Eu^{3+}, Sm^{3+}, Tb^{3+}/Eu^{3+}, Tb^{3+}/Sm^{3+}) as a multi-color emitting phosphor for UV w-LEDs. *Dalton Transactions* **2018,** *47* (20), 6995-7004.
15. Back, M.; R. Marin; Franceschin, M.; Hancha, N. S.; Enrichi, F.; Travea, E.; Polizziad, S., Energy transfer in color-tunable water-dispersible Tb–Eu codoped CaF_2 nanocrystals. *Journal of Materials Chemistry C* **2016,** *4*, 1906--1913.
16. Wang, L.; Yang, Z.; Li, Y.-F.; Yang, R.; Dai, Z.; Hu, S.; Sun, L.; Tong, Y., Fluorescence resonance energy transfer of CaF_2: Eu^{2+}, Tb^{3+} applied to dye-sensitized solar cells. *Spectrochimica Acta Part A: Molecular and Biomolecular Spectroscopy* **2018,** *202*, 76-80.
17. Zhu, H.; Qian, B.; Zhou, X.; Song, Y.; Zheng, K.; Sheng, Y.; Zou, H., Tunable luminescence and energy transfer of Tb^{3+}/Eu^{3+} co-doped cubic $CaCO_3$ nanoparticles. *Journal of Luminescence* **2018,** *203*, 441-446.
18. Bartolome, E.; Bartolome, J.; Arauzo, A.; Luzon, J.; Cases, R.; Fuertes, S.; Sicilia, V.; Sanchez-Cano, A. I.; Aporta, J.; Melnic, S.; Prodius, D.; Shova, S., Heteronuclear $\{Tb_xEu_{1-x}\}$ furoate 1D polymers presenting luminescent properties and SMM behavior. *Journal of Materials Chemistry C* **2018,** *6* (19), 5286-5299.
19. Miyata, K.; Konno, Y.; Nakanishi, T.; Kobayashi, A.; Kato, M.; Fushimi, K.; Hasegawa, Y., Chameleon luminophore for sensing temperatures: control of metal-to-metal and energy back transfer in lanthanide coordination polymers. *Angewandte Chemie* **2013,** *52* (25), 6413-6.
20. Li, J.; Zhang, Z.; Li, X.; Xu, Y.; Ai, Y.; Yan, J.; Shi, J.; Wu, M., Luminescence properties and energy transfer of $YGa_{1.5}Al_{1.5}(BO_3)_4:Tb^{3+},Eu^{3+}$ as a multi-colour emitting phosphor for WLEDs. *Journal of Materials Chemistry C* **2017,** *5* (25), 6294-6299.
21. Bettinelli, M.; D Flint, C., Non-resonant energy transfer between Tb^{3+} and Eu^{3+} in the cubic hexachloroelpasolite crystals $Cs_2NaTb_{1-x}Eu_xCl_6$ (x=0.01-0.15). *Journal of Physics: Condensed Matter* **1990,** *2* (42), 8417.
22. de Lill, D. T.; de Bettencourt-Dias, A.; Cahill, C. L., Exploring lanthanide luminescence in metal-organic frameworks: synthesis, structure, and guest-sensitized luminescence of a mixed europium/terbium-adipate framework and a terbium-adipate framework. *Inorganic chemistry* **2007,** *46* (10), 3960-3965.
23. Berry, M. T.; May, P. S.; Hu, Q., Calculated and observed $Tb^{3+}(^5D_4) \rightarrow Eu^{3+}$ electronic energy transfer rates in $Na_3[Tb_{0.01}Eu_{0.99}(oxydiacetate)_3]$-$2NaClO_4$-$6H_2O$. *Journal of Luminescence* **1997,** *71* (4), 269-283.

24. Bettinelli, M.; Piccinelli, F.; Speghini, A.; Ueda, J.; Tanabe, S., Excited state dynamics and energy transfer rates in $Sr_3Tb_{0.90}Eu_{0.10}(PO_4)_3$. *Journal of Luminescence* **2012,** *132* (1), 27-29.
25. Carrasco, I.; Bartosiewicz, K.; Nikl, M.; Piccinelli, F.; Bettinelli, M., Energy transfer processes in $Ca_3Tb_{2-x}Eu_xSi_3O_{12}$ (x=0–2). *Optical Materials* **2015,** *48*, 252-257.
26. Bao, G.; Wong, K.-L.; Jin, D.; Tanner, P. A. A stoichiometric terbium-europium dyad molecular thermometer: energy transfer properties, *Light: Science & Applications*, 7, (2018) 96 (1-10).
